# A Rare Case of Pulmonary Artery Intimal Sarcoma Diagnosed Antemortem by Endoscopic Ultrasound-Guided Fine-Needle Aspiration

**DOI:** 10.7759/cureus.112616

**Published:** 2026-07-13

**Authors:** Sunjun Huh, Yuya Yamamoto, Toshihiro Shirai

**Affiliations:** 1 Postgraduate Clinical Training Center, Keio University Hospital, Tokyo, JPN; 2 Department of Respiratory Medicine, Shizuoka General Hospital, Shizuoka, JPN

**Keywords:** eus-fna, intimal sarcoma, primary pulmonary artery sarcoma, primary pulmonary artery sarcoma (ppas), pulmonary artery sarcoma

## Abstract

Primary pulmonary artery sarcoma (PPAS) is a rare malignancy often misdiagnosed as pulmonary thromboembolism. Antemortem diagnosis is often difficult because of the tumor’s deep intravascular location and challenges in obtaining tissue samples. We report a 72-year-old man with pulmonary artery intimal sarcoma diagnosed antemortem using endoscopic ultrasound-guided fine-needle aspiration (EUS-FNA). Contrast-enhanced computed tomography demonstrated a heterogeneously enhancing intraluminal pulmonary artery lesion, and positron emission tomography/computed tomography revealed intense fluorodeoxyglucose uptake (SUVmax 18.4). Histopathological examination showed atypical spindle-shaped cells positive for vimentin and focally positive for α-smooth muscle actin, consistent with intimal sarcoma. The patient received radiation therapy and doxorubicin-based chemotherapy but died 25 weeks after admission. Consistent with previous reports, this case illustrates the aggressive clinical course and diagnostic challenges of PPAS. It also highlights the importance of considering PPAS in patients with atypical pulmonary embolism and demonstrates the utility of EUS-FNA as a minimally invasive approach for obtaining a definitive diagnosis of centrally located pulmonary artery tumors.

## Introduction

Primary pulmonary artery sarcoma (PPAS) is an exceedingly rare and highly aggressive malignancy arising from the pulmonary artery, with an estimated incidence of 0.001-0.003% [1,2]. It is broadly classified into intimal sarcoma, arising from the vascular intima, and mural sarcoma, originating from the vessel wall. Intimal sarcoma is the most common histological subtype and is characterized by rapid progression and poor prognosis [1,3,4].

Because PPAS frequently presents with nonspecific symptoms, such as dyspnea, chest pain, cough, and hemoptysis, it is commonly misdiagnosed as pulmonary thromboembolism (PTE) [3,4]. Furthermore, imaging findings may overlap considerably with thromboembolic disease, often resulting in delayed diagnosis and treatment [4]. Nearly half of PPAS cases are initially misdiagnosed as pulmonary embolism, with many of them receiving inappropriate thrombolytic and/or anticoagulation therapy [4]. Antemortem diagnosis remains difficult because of the tumor's deep intravascular location and the technical challenges associated with obtaining tissue samples. Consequently, many cases are diagnosed only at surgery or autopsy [3,4].

Herein, we report a rare case of pulmonary artery intimal sarcoma successfully diagnosed antemortem using endoscopic ultrasound-guided fine-needle aspiration (EUS-FNA), highlighting the diagnostic utility of minimally invasive tissue acquisition in suspected pulmonary artery malignancy.

## Case presentation

A 72-year-old man with a history of myocardial infarction and cholangitis presented with a six-month history of exertional dyspnea and chest discomfort. He had previously smoked 20 cigarettes daily for 45 years and consumed approximately 200 mL of beer per day. His medications included prasugrel, rosuvastatin, vonoprazan, and carvedilol.

**Figure 1:**
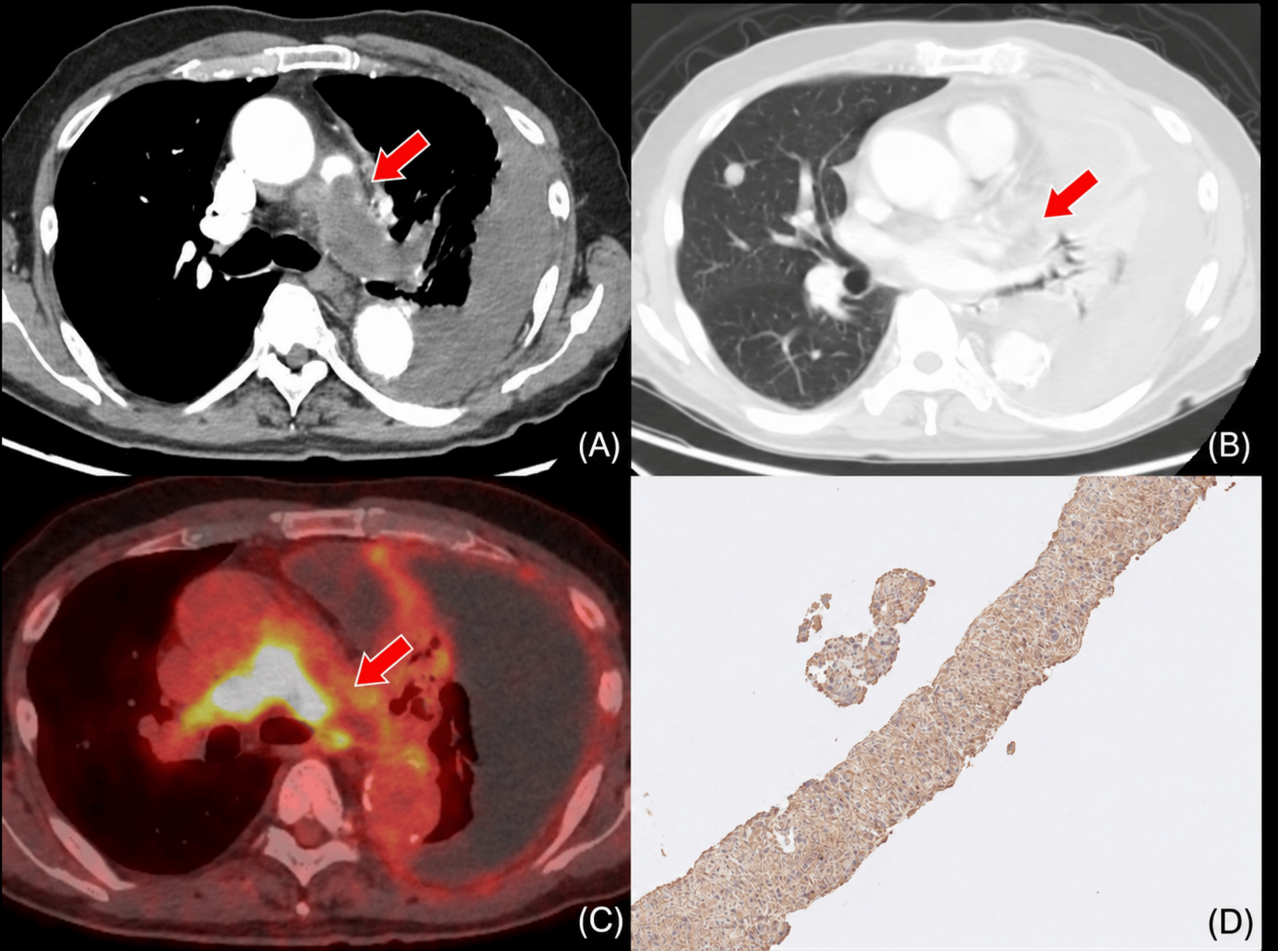
Radiologic and histopathologic findings of pulmonary artery intimal sarcoma (A, B) Contrast-enhanced chest CT demonstrating a heterogeneously enhancing intraluminal mass within the left pulmonary artery. (C) PET/CT showing intense FDG uptake within the lesion (SUVmax 18.4). (D) EUS-FNA specimen demonstrating spindle-shaped atypical tumor cells positive for vimentin immunostaining, consistent with intimal sarcoma. PET/CT: positron emission tomography/computed tomography; FDG: fluorodeoxyglucose; SUVmax: maximum standardized uptake value; EUS-FNA: endoscopic ultrasound-guided fine-needle aspiration

Because his symptoms progressively worsened, he initially visited a local hospital, where contrast-enhanced computed tomography (CT) suggested left pulmonary artery thromboembolism. He was subsequently referred to our institution for further evaluation and management. Repeat contrast-enhanced CT demonstrated an intraluminal filling defect within the left pulmonary artery with heterogeneous contrast enhancement (Figures 1A, 1B), an atypical feature for thromboembolism. The CT also demonstrated a left pleural effusion and associated left lung atelectasis, findings that are uncommon in uncomplicated pulmonary thromboembolism. Positron emission tomography/computed tomography (PET/CT) further revealed intense fluorodeoxyglucose uptake within the lesion, with a maximum standardized uptake value (SUVmax) of 18.4 (Figure 1C), raising strong suspicion for a malignant process rather than thromboembolic disease.

On admission, his temperature was 37.6 °C, blood pressure was 115/65 mmHg, pulse rate was 96 beats/min, respiratory rate was 16 breaths/min, and oxygen saturation was 94% on room air. Physical examination revealed diminished breath sounds over the left lung field without crackles or cardiac murmurs. No lower extremity edema or cervical lymphadenopathy was observed.

Laboratory examination showed leukocytosis (white blood cell count, 11,600/μL; reference range, 3,300-8,600/μL), anemia (hemoglobin, 9.0 g/dL; reference range, 13.7-16.8 g/dL), thrombocytosis (platelet count, 47.9 × 10^4^/μL; reference range, 15.8-34.8 × 10^4^/μL), elevated C-reactive protein (21.6 mg/dL; reference range, <0.14 mg/dL), and mildly elevated D-dimer levels (3.4 μg/mL; reference range, <1.0 μg/mL). Tumor markers, including carcinoembryonic antigen (reference range, <5.0 ng/mL), CYFRA (reference range, <3.5 ng/mL), and pro-gastrin-releasing peptide (reference range, <81 pg/mL), were within normal limits.

Given the intravascular location of the lesion and the difficulty of obtaining tissue by conventional bronchoscopic approaches, EUS-FNA was performed. Histopathological examination demonstrated atypical spindle-shaped tumor cells. Immunohistochemical staining showed positivity for vimentin (Figure 1D) and focal positivity for α-smooth muscle actin (α-SMA), whereas AE1/AE3, epithelial membrane antigen, desmin, and S100 were negative. These findings were consistent with pulmonary artery intimal sarcoma.

Following diagnosis, the patient received palliative radiation therapy to the left pulmonary artery lesion (17 Gy in 2 fractions) to relieve symptoms, followed by doxorubicin-based chemotherapy. Despite treatment, the disease progressed rapidly, and the patient ultimately died 25 weeks after admission under best supportive care.

## Discussion

PPAS is a highly aggressive malignancy associated with poor clinical outcomes. Complete surgical resection remains the only potentially curative treatment and has been associated with markedly prolonged survival compared with incomplete resection or nonsurgical management [5]. However, because diagnosis is often delayed, many patients present with advanced or unresectable disease. Approximately 50% of patients have pulmonary metastases and 16% have distant metastases at the time of diagnosis [6].

Differentiating PPAS from pulmonary thromboembolism is clinically important yet challenging. Both conditions may present with dyspnea, chest pain, and cough. However, several imaging findings may suggest PPAS rather than thromboembolism, including unilateral central pulmonary artery involvement, lobulated or expansile intraluminal masses, heterogeneous contrast enhancement on CT, and marked fluorodeoxyglucose (FDG) uptake on PET/CT. In contrast, thromboembolic lesions usually lack enhancement and typically demonstrate lower FDG uptake. In the present case, the strong contrast enhancement and high SUVmax of 18.4 were key findings that prompted further investigation for malignancy (Table 1). In addition, the presence of pleural effusion and left lung atelectasis in our patient was atypical for uncomplicated pulmonary thromboembolism and further supported the possibility of an underlying malignant process.

**Table 1:** Differential features between primary pulmonary artery sarcoma and pulmonary thromboembolism The information was adapted from previous reports [6-8]. PET/CT: positron emission tomography/computed tomography; SUVmax: maximum standardized uptake value

	Primary pulmonary artery sarcoma	Pulmonary thromboembolism
Clinical symptoms	Cough, chest pain, dyspnea	
D-dimer	Normal to elevated	Elevated
CT	Often central	Varies from central to peripheral
	Mass-like or lobulated shape; proximal portion appears bulging; heterogeneous with contrast enhancement	Proximal portion appears flat or cup-shaped; no contrast enhancement
T2 signal intensity	High	Low
Mean SUVmax on PET/CT	7.3	2.4

The definitive diagnosis of PPAS requires histopathological confirmation. However, obtaining tissue samples from pulmonary artery lesions is technically difficult and potentially hazardous. Previous reports have described diagnosis through surgical biopsy, endovascular catheter biopsy, or autopsy. In our case, EUS-FNA enabled minimally invasive tissue acquisition and successful antemortem diagnosis. This approach may represent a valuable diagnostic option in carefully selected patients with centrally located pulmonary artery tumors adjacent to the esophagus.

Although the prognosis of unresectable PPAS remains poor, earlier diagnosis may facilitate timely multidisciplinary treatment planning, including surgical evaluation, systemic chemotherapy, and radiotherapy. To our knowledge, reports describing antemortem diagnosis of pulmonary artery intimal sarcoma using EUS-FNA remain extremely limited. Our case, therefore, expands the existing literature by demonstrating that EUS-FNA can provide a safe and minimally invasive means of obtaining diagnostic tissue from centrally located pulmonary artery tumors that are difficult to access using conventional bronchoscopic or percutaneous techniques. This case also reinforces the importance of integrating characteristic CT and PET/CT findings with minimally invasive tissue sampling to distinguish PPAS from pulmonary thromboembolic disease and establish an early definitive diagnosis.

## Conclusions

Primary pulmonary artery intimal sarcoma is a rare and aggressive malignancy that can closely mimic pulmonary thromboembolism, often resulting in delayed diagnosis. In patients with atypical pulmonary artery lesions demonstrating heterogeneous contrast enhancement and increased FDG uptake, PPAS should be considered in the differential diagnosis. This case highlights the value of endoscopic ultrasound-guided fine-needle aspiration as a minimally invasive approach for obtaining a definitive histopathological diagnosis of centrally located pulmonary artery tumors when conventional biopsy methods are challenging. Early recognition and tissue confirmation may facilitate timely multidisciplinary treatment planning.
